# Notes and News

**Published:** 1952-04

**Authors:** 


### *Notes and News*

### THE ROYAL COLLEGES

*F.R.C.O.G.* Professor G. G. Lennon.

*D.C.H.* Dorothy M. Pierce.

### THE CLINICAL SOCIETY OF BATH

*May 2nd.* The Annual General Meeting.

*May 3rd.* The Annual Dinner, Fortt's Restaurant, Bath.

All meetings are held at the Royal United Hospital, Bath, at 8.30 p.m. Country membership of the Society is open to male practitioners in any part of the South West and the annual subscription is 10s. 6d. Particulars may be obtained from the Hon. Secretary, Mr. A. Daunt Bateman of 3 The Circus, Bath.

